# Observations on the Medical Platform

**Published:** 1854-01

**Authors:** 


*Observations on the Medical Platform.*—This is the title of an introductory lecture, delivered at Ann Arbor, by Prof. J. Adams Allen, a gentleman who holds several professorships—permanent and acting—in the medical school located at that place.

In complying with the request of a correspondent to notice this production, we do not feel at liberty to dismiss it with the usual words of vague commendation bestowed on introductories. On the other hand, in attempting to analyze it, and otherwise to perform the usual offices of the critic, we are placed in a somewhat unpleasant position. First let us inform our readers of the intent of the lecture.

So far as we can discover, it foreshadows the inauguration of some new movement in medicine—some comprehensive reform, which is to issue from the peninsular school of the prophets—and is to accomplish great purposes in some unexplained way. That is, if the lecture has any meaning, it means this.

Now we are aware of a propensity, which is endemic at the west, to a certain style of oratory, vulgarly called, "high falutin." It is possible that this may be the proximate cause of this overflow. Everything is attacked. We are told that legitimate medicine is a very rickety frame work; that medical books are collections of errors; and medical ethics and codes are arrant humbugs; that there is an outward pressure upon the profession that will assuredly crush it; that *sauve qui peut* is the wise man's game; and that, if the temple of Esculapius is to be torn down, we may as well side with the movement, and join in what will be inevitable

We quote: "What matters it that Hippocrates, and Aristotle, and Plato, Galen, Rhazes, Aviceuna, Aetius and Celsus, Harvey and Boerhaave, Hunter, Broussais, Sydenham and Rush, have aided in the erection of this pyramid, if all their labors have converged to an apex upon which our modern temple finds insecure support? It is not men, nor time, nor circumstance, which evoke of necessity the clear and warrantable body of truth. For truth as such is utterly independent of the media through which it is transmitted to us; our acceptance or explanation of it, are in nowise its measure or its proof.

"Medical veterans may rage with awful front, and as they exhibit their honorable scars and mutilated limbs, may denounce the ingratitude and monstrous perversion of the times, but this outlay of vital force may as well be husbanded for other purposes."

And again: "What matters it that mest grave and reverend doctors in the law of medicine, in solemn conclave declare, 'These are the highest deductions of human reason; these, the strongest teachings of experience; these, the ultimates upon which the human mind must rest?' So other arts and other sciences have been enclosed, the outward pressure has burst the feeble barriers, and Antiquity, and Precedent, and Opinion, and the assumed Results of Experience have been commingled in confusion worse confounded, until Order, Heaven's first law, has called the living productive forms of eternal truth out of the wreck of foolish wisdom."

*"Ridentem dicere verum."*

What is to happen? We are in a most painful condition of solicitude and uncertainty. In the general crush which must follow this bursting of the barriers from outward pressure, may we and ours be preserved!

Seriously, we are weary of Jeremiads and diatribes, directed by medical men at medical science. Just at present, no profession or occupation is accomplishing more in the paths of true progress, or doing more every-day good to humanity in its ministrations, than is medicine. It is a mistake to suppose that the profession is system-ridden. Its organization has for its vital principle the freedom of opinion of the individual practitioner; and there are no creeds or doctrines to which he is bound to subscribe. If legitimate medicine is at a discount in Michigan, such prelections as this will furnish very specious arguments to its enemies. But perhaps it is necessary in a government school, to adapt its teachings to the pleasure of the appointing power—the sovereign people.

We find in a Detroit newspaper a criticism, which has in some measure suggested this notice, which we close with a brief quotation from the aforesaid criticism:

"The Dr. has evidently solved that grand Pilate-question, 'What is truth?' entirely to his own satisfaction, and has come to the conclusion that, my *thinking* a thing to be true, makes it true; and that this is the only kind of truth at which we can arrive or which can exist. This is a very purifying and ennobling doctrine for a teacher of youth in the University of Michigan to inculcate before a class of the hopeful youth of that establishment. It is calculated to increase very considerably our respect for, and confidence in, state education, and to diminish our fears that it will be productive in the end of an all-disastrous and all-engulphing atheism.

"We regret that our space is too valuable to allow us to devote more of it to the Dr. and his lucubrations. The style of the lecture is entirely too magnificent to admit of criticism; we should as soon think of making a critical examination of the crown jewels used by an actor in personating King Richard or King Lear. The Dr.'s phrase 'stand point of view,' seems to us to savor somewhat of redundancy, and reminds us of a classmate of our own who with a heart full of Paschal joy, exclaimed one Easter morning: Resurrexit—*est!* Some of the Professor's puns, too, are rather excruciating—as when he talks about 'ponderous *(not tombs,* but) *tomes.*' These, however, are but minor blemishes—mere spots on the sun." S. B. H.

